# Supplementary material for: Glucocorticoid and dietary effects on mucosal microbiota in canine inflammatory bowel disease
Source: PLoS One. 2019 Dec 30;14(12):e0226780. doi: 10.1371/journal.pone.0226780 (PMC6936794; doi:10.1371/journal.pone.0226780)
Supplement: Attachment — Submitted filename: Response to reviewers_Oct_2019.docx [file pone.0226780.s005.docx]

**RESPONSE TO REVIEWERS (PONE-D-19-23630)**:

My specific critiques are below:

- Though the authors use the term ‘spatial distribution of microbiota’ in terms of mucosal compartments, however do not provide any analyses of the difference in distribution between ileum and colon, which were used for FISH analysis. The authors should clarify this and provide data obtained from ileal and colonic samples, since these two intestinal regions have been shown to have different composition of microbiota in various experimental animal species and also in humans

***Intestinal tissues (endoscopic biopsies of ileum and colon) for FISH analysis were combined on	glass slides for ease of hybridization and consistency of interpretation by a single operator. In	this manner, a single slide would contain both ileal and colonic tissues (3-5 samples of each	tissue) in which the operator would perform 10 individual bacterial counts in 4 separate	endoscopic biopsies. While care was taken to accurately distinguish between 2 ileal versus 2	colonic	mucosal samples for quantitative analysis, a clear distinction between these tissues	could not always be determined. Therefore, mucosal bacterial counts for each animal were	recorded as a cumulative total of bacteria observed within ileal/colonic intestinal segments	rather than separate bacterial counts for the ileum and separate bacterial counts for the	colon, respectively. We report a similar study design for FISH analysis in a recent report.^1^***

***^1^White, et al. Randomized, controlled trial evaluating the effect of multi-strain probiotic on the	mucosal microbiota in canine idiopathic inflammatory bowel disease. Gut microbes. 2017;	8(5):451-66.***

***We have now modified the methods section to more accurately reflect how mucosal bacteria	in endoscopic samples were counted across intestinal segments.***

- Line 381 in discussion: The authors indicate that there’s a change in the distribution and composition of mucosal microbiota, which is misleading. The authors have only looked at five bacteria and their differential expression in the experimental groups. The authors should change the sentence and not extrapolate the observation to differential microbial composition or intestinal site distribution.

***Agreed and now modified in discussion.***

- The authors should speculate and discuss why glucocorticoids may alter the differential expression of the three bacteria (line 383)

***Speculation is required here since there is an absolute paucity of information on how GCs may	modulate gut mucosal bacterial populations in health or disease.* *We now state*:**

***It is clear that dysbiosis of the intestinal bacteria is both a cause and consequence of IBD. As	antibiotics and probiotics have only modest effects in managing patients with IBD, many	clinicians now opt for a top-down therapeutic approach using biologics and/or	immunosuppressive drugs, such as glucocorticoids, to induce clinical remission. Only few	studies show that GCs (or any other immunosuppressive drug for that matter) can exert	favorable effects in the fecal microbiome of IBD affected individuals. However, there are no	studies (beyond the present report) which describe how or why GCs selectively alter the	mucosal microbiota in IBD. It is also possible that other components of the gut	microbiome	(i.e., fungi and viruses) are influenced by GC administration and contribute to beneficial	modulation of resident bacterial populations as observed in this study.***

- Just for clarity: Were all the biopsies done at the end of 8 weeks of diet and treatment?

***Yes, IBD dogs for repeat GI endoscopy were re-examined during treatment within 1-3 days of	diet/drug trial completion.***

**Review Comments to the Author**
Reviewer #1: The authors have used two major techniques (IHC and FISH) to determine the effect of glucocorticoid and diet on mucosal microbiota in canine IBD. The manuscript is well written and has very few typographical errors. The authors have provided the raw data that was used for data analysis.

***There are no specific comments to address.***

Reviewer #2: Glucocorticoids are commonly administered to reduce mucosal inflammation and gastrointestinal signs.
The study evaluated the effects of diet and oral prednisone on the spatial distribution of mucosal bacteria in IBD dogs. Whether or not GCs beneficially modulate the mucosal microbiota to reduce intestinal inflammation in dogs with IBD has not been systematically evaluated using fluorescence in situ hybridization. The current study should be interesting as it will provide new information in treating IBD dogs and understand the association of inflammation and microbiome. However, the authors need address the following concerns from the reviewer:

1. The sample size is relatively small (IBD dog 8, health control 15). Please clarify the IBD stages of each dog. The focus of the analysis should be the IBD dogs before and after treatment.

***Agreed that the focus is on the IBD dogs pre- versus post-treatment. Regarding the various clinical stages of IBD dogs at diagnosis (pre-treatment), 3 dogs with IBD had severe clinical disease activity (i.e., CIBDAI scores ≥ 9) and 5 dogs with IBD had moderate disease activity (i.e., CIBDAI scores 6-8). At trial completion, all 8 dogs with IBD were in full clinical remission with a mean post-treatment CIBDAI score of 1. These new data are now included in the revised manuscript. Good suggestion.***

2. Please clarify that E-cadherin is not a tight junction protein.

***Thanks for pointing out that there is a difference between the different classes of apical junction proteins (AJPs), which include the tight junction proteins (occludin, claudin family, and ZO-1) and the adherens junction (AJ) protein, E-cadherin. To avoid mis-characterization of the protein classes we evaluated using IHC, we have now corrected the manuscript to state AJPs and their respective proteins rather than TJPs. Good suggestion and sorry for the confusion. We have now updated the discussion section appropriately.***

3. The Results are poorly written. More details are needed.

***Thank you for this recommendation and we have now expanded the results section regarding patient demographics and FISH analysis to include more detail and clarity as requested.***

4. The Figure Legends should be separated from the Results.

***Now separated as requested.***

5. It is helpful to extend the Discussion and relate the current study to human IBD, which is better established.

***Good suggestion and additional information now provided for the reader. That said, there is no information***
