# Supplementary material for: Glucocorticoid and dietary effects on mucosal microbiota in canine inflammatory bowel disease
Source: PLoS One. 2019 Dec 30;14(12):e0226780. doi: 10.1371/journal.pone.0226780 (PMC6936794; doi:10.1371/journal.pone.0226780)
Supplement: Attachment — Submitted filename: Reviewer Comments-PONE-D-19-23630.docx [file pone.0226780.s004.docx]

Overall critique:

This paper describes the effects of glucocorticoid and diet on mucosal microbiota in canine IBD. The authors have used IHC and FISH to determine the differences in the expression of tight junction proteins and microbiota, respectively. The authors have provided an observational analysis of the IHC and FISH data without any functional or mechanistic alterations in IBD and thereafter. It is appreciable that the authors point to the limitations of their study in the discussion, which could be easily noted.

My specific critiques are below:

- Though the authors use the term ‘spatial distribution of microbiota’ in terms of mucosal compartments, however do not provide any analyses of the difference in distribution between ileum and colon, which were used for FISH analysis. The authors should clarify this and provide data obtained from ileal and colonic samples, since these two intestinal regions have been shown to have different composition of microbiota in various experimental animal species and also in humans
- Line 381 in discussion: The authors indicate that there’s a change in the distribution and composition of mucosal microbiota, which is misleading. The authors have only looked at five bacteria and their differential expression in the experimental groups. The authors should change the sentence and not extrapolate the observation to differential microbial composition or intestinal site distribution
- The authors should speculate and discuss why glucocorticoids may alter the differential expression of the three bacteria (line 383)
- Just for clarity: Were all the biopsies done at the end of 8 weeks of diet and treatment?
